# Sex and *APOE* ε4 Allele Shape Behavioral and Epigenetic Susceptibility to Prenatal Chlorpyrifos Exposure in Mice

**DOI:** 10.3390/toxics14030212

**Published:** 2026-02-28

**Authors:** Judit Biosca-Brull, Laia Guardia-Escote, Jordi Blanco, Maria Cabré, Pia Basaure, Fernando Sánchez-Santed, José L. Domingo, Maria Teresa Colomina

**Affiliations:** 1 Universitat Rovira i Virgili, Department of Biochemistry and Biotechnology, 43007 Tarragona, Spain; judit.biosca@urv.cat (J.B.-B.); maria.cabre@urv.cat (M.C.); 2 Universitat Rovira i Virgili, Research Group in Neurobehavior and Health (NEUROLAB), 43007 Tarragona, Spain; jordi.blanco@urv.cat (J.B.); pia.basauregarcia@gmail.com (P.B.); 3 Grup de Recerca en Toxicologia (GRET) and Toxicology Unit, Department of Pharmacology, Toxicology and Therapeutical Chemistry, Faculty of Pharmacy, University of Barcelona, 08028 Barcelona, Spain; laia.guardia@ub.edu; 4 Universitat Rovira i Virgili, Department of Basic Medical Sciences, 43201 Reus, Spain; 5 Department of Psychology and Research Center for Well-Being and Social Inclusion (CIBIS), University of Almeria, 04120 Almeria, Spain; fsanchez@ual.es; 6 Universitat Rovira i Virgili, Laboratory of Toxicology and Environmental Health, The Center for Environmental, Food and Toxicological Technology (TecnATox), 43201 Reus, Spain; joseluis.domingo@urv.cat; 7 Universitat Rovira i Virgili, Department of Psychology and Research Center for Behavior Assessment (CRAMC), 43007 Tarragona, Spain

**Keywords:** apolipoprotein E, chlorpyrifos, organophosphates, learning and memory, anxiety, DNA methylation

## Abstract

Pesticides are essential for modern agriculture but raise concerns about potential neurodevelopmental consequences, leading to bans in some countries. This study aimed to investigate the long-term effects of prenatal exposure to chlorpyrifos (CPF) on behavior and DNA methylation, considering genetic susceptibility via the apolipoprotein E (*APOE*) genotype. Pregnant mice—C57BL/6J and those carrying human *APOE* ε3 or ε4 alleles—were orally exposed to 0 or 1 mg/kg/day of CPF from gestational day 12 to 18. Adult offspring underwent light and dark and Morris water maze tests to assess anxiety-like behavior and spatial learning and memory. Then, hippocampal samples were collected to assess DNA methylation. Results indicated that body weight was lower in females and CPF-treated mice. C57BL/6J males spent less time in the light compartment, worsened by CPF. In contrast, within *APOE* genotype ε4 carriers spent more time in the light compartment, with CPF increasing male activity. Moreover, long-term retention was impaired in both male and female apoE4 mice prenatally exposed to CPF. DNA methylation analysis revealed sex-dependent differences, with hypomethylation in the CPF-treated male hippocampus. These findings highlight how pesticides and genetic factors interact, affecting neurobehavioral development, and explore the potential impact of CPF on DNA methylation.

## 1. Introduction

Pesticides are indispensable for agricultural production. For many years, they have been used to control corn yields and pests such as ticks, fleas, termites, chinch bugs, aphids, or mosquitoes [1]. Organochlorine insecticides were the first class of pesticides to be used, in particular, dichlorodiphenyltrichloroethane (DDT). Nevertheless, the evaluation of toxicity and ecotoxicity of DDT revealed its potential to cause serious adverse environmental and human health effects [2].

Since the use of the popular DDT was restricted in 1972, organophosphates (OPs) have been used to control insects in households and agricultural fields. However, it has been observed that persistent use of these pesticides can affect non-target organisms, posing risks to both humans and animals [3]. The best-known OP compound is chlorpyrifos (CPF) [4,5]. The main neurotoxic effect of CPF, and its principal active metabolite CPF-oxon, is the inhibition of the enzyme acetylcholinesterase (AChE). AChE is responsible for the breakdown of acetylcholine (ACh) in the synaptic cleft in the central nervous system (CNS) and at the neuromuscular or neuro-glandular junctions of the peripheral nervous system. Blockade of the active site of AChE by CPF or CPF-oxon and its subsequent inhibition leads to an accumulation of ACh at synapses, resulting in cholinergic hyperstimulation [6,7,8].

The lipophilic properties of CPF allow it to cross the placenta and be absorbed by fetal tissues, including the developing brain. In addition, it can accumulate in breast milk and be transferred to infants or neonates [9,10,11]. Therefore, exposure to CPF could occur before the CNS reaches full maturity, a time of high risk for toxic effects of the pesticide [12,13,14]. Most experimental studies assessing prenatal exposure to CPF have employed doses ranging from 2.5 to 6 mg/kg, which often results in clear neurodevelopmental alterations [15,16]. However, several studies have shown that lower doses—at levels that do not produce detectable inhibition of AChE—can also lead to adverse outcomes [17,18,19,20,21,22,23,24]. These lower exposures are particularly relevant, as they are more representative and allow for the study of the subtle but potentially long-lasting effects of CPF. For instance, exposure to low doses of CPF (1 mg/kg/day) during neurogenesis resulted in social deficits in adolescent C57BL/6J mice [25] and alterations in rat communication by increasing the latency to emit the first call and reducing the total number of calls [26]. In addition, Levin et al. [27] and Icenogle [28] found that rats prenatally exposed to 1 or 5 mg/kg/day of CPF exhibited alterations in working and reference memory in a figure-8 maze and 16-arm radial maze during adolescence and adulthood. This indicates that exposure to CPF during fetal development causes behavioral impairments that persist to adulthood. Due to these adverse effects, CPF was banned for indoor use in January 2001 [29,30,31] and was fully prohibited by 2021 [32,33]. Despite this, the population continues to be exposed to the pesticide as a consequence of its use in developing countries. Consequently, CPF remains in the environment and can be spread around the world by routes such as ocean currents [34].

Behavioral adverse effects are not only produced by exposure to environmental contaminants, but genetics also play a key role in the vulnerability to these effects [35]. The apolipoprotein E (*APOE*) gene is polymorphic and, in humans, codes for three main isoforms (apoE2, apoE3, and apoE4), which are distributed differently throughout the population (apoE3 > apoE4 > apoE2) [36,37,38,39,40]. The primary function of APOE is to transport and deliver lipids between tissues and cells, maintaining lipid homeostasis [38,41]. This protein plays a crucial role during early stages of development, when cholesterol and other lipids are needed for processes such as neurogenesis, synaptogenesis, or myelination. However, the distribution and elimination of lipids depends on the type of APOE isoform, indirectly affecting cognition and behavior [42,43]. In humans, homozygous carriers of the *APOE* ε4 allele are at an increased risk of developing Alzheimer’s disease (AD), but it appears to confer cognitive advantages in young adults [44,45]. In the same line, animal studies have reported that the *APOE* genotype, and specifically the ε4 allele, is associated with learning and memory deficits and in increased anxious, stressful, and depressive behavior [46,47,48,49].

Different levels of vulnerability to the environmental toxicant CPF were also observed in relation to the apoE isoform [50]. Postnatal exposure to 1 or 3 mg/kg/day of CPF increased impulsive and compulsive behaviors, as well as the preference for the social stimulus in *APOE* ε4 carriers, while performance in learning and memory tasks was worse, especially in females [51,52,53].

In addition to genetic factors, the epigenetic mechanisms involved in the early embryonic development of mammals are considered to be highly vulnerable to environmental toxicants [54]. Although research on DNA methylation in relation to *APOE* allele variations and CPF exposure remains limited, emerging evidence is beginning to shed light on this area. In this line, Guardia-Escote et al. [55] examined the DNA methylation in hypothalamic genes related to feeding regulation in homozygous mice for human *APOE* ε3 and ε4 alleles that were postnatally exposed to CPF. The study revealed notable sex- and genotype-dependent effects that vary by gene and specific DNA sites.

Despite growing evidence on the effects of the *APOE* genotype and postnatal exposure to CPF separately, their potential interactions, the influence of sex-specific differences and underlying regulatory mechanisms during early developmental stages remain poorly understood. Therefore, in this study, we focused on investigating mechanisms related to the long-term effects of prenatal CPF exposure during fetal development. Specifically, we studied the adverse effects of CPF on anxiety-like behaviors and spatial learning and memory. This investigation was based on the strong association between the *APOE* ε4 allele and AD. Additionally, we conducted an exploratory analysis of DNA methylation in the hippocampus of a transgenic mouse model carrying the *APOE3* and *APOE4* genotypes to assess the potential of CPF to act as an epigenetically active compound.

## 2. Materials and Methods

### 2.1. Animals

Adult male and female mice were used in this study. The C57BL/6J mice were obtained from Charles River Laboratories (Barcelona, Spain), while the human apoE3- and apoE4-target replacement (TR) homozygote mice were obtained from Taconic Europe (Lille Skensved, Denmark). The apoE-TR model has a C57BL/6NTac background and was generated by replacing the murine *ApoE* gene with the human *APOE* ε3 or ε4 allele, without altering any endogenous regulatory sequence [56]. After one week in quarantine, one male and two females of the same strain were mated for 3 h. The animals were then separated, and the presence of a vaginal plug indicated gestational day (GD) 0.

All the mice were housed in plastic cages containing a minimum of two and a maximum of five animals. On GD 12, pregnant females were housed individually and randomly assigned to one of the two treatment groups (control (CNT) or CPF). Delivery day was assigned as postnatal day (PND) 0. Litters with less than four pups were excluded. A total of four apoE3 litters, four apoE4 litters and five C57BL/6J litters were excluded in the study. All animals had *ad libitum* access to food (SAFE^®^ A04 diet, Panlab, Barcelona, Spain) and tap water and were maintained in a 12 h light/dark automatic cycle (light on from 8:00 a.m. to 8:00 p.m.), at a controlled temperature (22 ± 2 °C) and humidity (50 ± 10%).

### 2.2. Treatment and Experimental Design

To assess the effect of prenatal exposure to low doses of CPF in adults, pregnant female mice were fed a diet containing 0 or 1 mg/kg/day between GD 12 and 18. This exposure window was selected because in this period, active neurogenesis, neuronal migration, and early hippocampal formation occur, making this developmental window particularly sensitive to environmental insults [57]. The dose was selected because it is below the threshold for cholinergic effects [8]. Litter characteristics and postnatal outcomes were previously described by Biosca-Brull et al. [58], confirming that the CPF dose used in this study does not induce reproductive toxicity. CPF (0,0-diethyl O-(3,5,6-trichloropyridin-2-yl) phosphorothioate) with 99.5% purity was obtained from Sigma-Aldrich (Madrid, Spain). The standard food was supplemented with 15 mg CPF/kg (Panlab, Barcelona, Spain) and adjusted to administer the required dose. Maternal body weight and food consumption were monitored daily to confirm the dosage administered.

**Figure 1:**
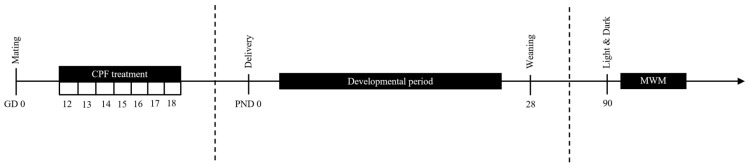
Schematic representation of the experimental design. Pregnant C57BL/6J and apoE-TR females received a diet containing either 0 or 1 mg/kg/day of CPF from GD 12 to 18. The day of birth was designated as PND 0. On PND 28, pups were separated from their dams and housed in plastic cages with a maximum of five animals per cage. At adulthood, anxiety-like behavior and spatial learning and memory were evaluated using the light and dark test and the Morris water maze (MWM) test, respectively.

Pups remained with their mothers in their home cage until they were 28 days old. Then, a maximum of two pups per litter and sex were separated into different cages according to strain, prenatal treatment, genotype, and sex for subsequent behavioral testing at three months of age (Figure 1). After weaning, body weight was measured on PND 60, before behavioral battery testing started on PND 90 and on days 5 and 8 of the Morris water maze (MWM) test. Table 1 shows the total number of animals used in this study.

### 2.3. Sample Collection

Twenty-four hours after the end of the behavioral tests, animals were deeply anesthetized with isoflurane delivered in room air until complete loss of responsiveness to external stimuli was confirmed. Once deep anesthesia was achieved, euthanasia was performed by cardiac puncture for blood collection. Plasma samples were obtained and organs were subsequently removed. The brain was rapidly extracted, and the hippocampus was dissected on ice. All samples were flash-frozen in liquid nitrogen and stored at −80 °C until biochemical analysis.

### 2.4. Behavioral Tests

At three months of age, male and female mice were tested to evaluate the long-term effects of CPF exposure. First, anxiety-like behavior was assessed using the light and dark test. Then, spatial learning and memory were evaluated using the MWM test. Before starting the test, mice were moved to the testing room and left undisturbed for 15 min.

### 2.5. Light and Dark Test

The light and dark apparatus consisted of a rectangular cage with two compartments: a dark compartment (24 × 21 × 21 cm) and a brightly illuminated compartment as the light compartment (24 × 21 × 21 cm). The animals were placed in the dark compartment and allowed to move freely between the two compartments for five minutes. To avoid potential biases related to olfactory cues, the apparatus was cleaned with 70% ethanol after each trial. The first time the mice entered the light compartment (latency), the number of entries (crossings) and the total time spent into the light compartment were manually recorded by a researcher.

### 2.6. Morris Water Maze Test

The water maze consisted of a circular pool measuring 1 m in diameter and 60 cm in height, divided into four virtual quadrants. One of those quadrants was designated as the target quadrant (TQ) and contained an escape platform, with a diameter of 10 cm, submerged 1 cm below the surface of the water. The walls surrounding the maze included black marks of different shapes used as visual extra-maze cues to help the animals to find the platform. To prevent the use of internal cues, we included an internal rotating wall inside the pool. This wall was rotated between trials to ensure spatial learning. We then defined four starting positions in the maze; these positions were changed between trials to prevent non-spatial trajectory learning. The water maze was divided into four phases: acquisition, retention, reversal learning, and visual. Before starting with the water maze test, mice were habituated to the pool by being placed in it (without the platform) for 45 s to freely swim and explore the space. During the acquisition phase, mice performed five sessions, which were distributed over five days. Each session consisted of four trials with an inter-trial interval of 30 min. Each trial finished either when the mice reached the platform or after 60 s. In the case that the animal did not find the platform, it was guided and remained there for 30 s. The retention was evaluated by two probe trials performed at 4 and 72 h after the last acquisition trial. These consisted of 60 s of free swimming after the escape platform had been removed. After probe trial 2, we began the reversal phase, which was performed in two sessions distributed over two days. This phase followed the same protocol as the acquisition phase, but in this case, the platform was located on the opposite side of the pool. To assess the retention of the new location of the platform, a reversal probe trial was performed 4 h after the last reversal session. The following day, the visual phase was assessed to evaluate the animals’ visual acuity, motor function, and general motivation to escape from the water. This phase was carried out in a single day and consisted of four consecutive trials. In contrast to acquisition and retention phases, the platform was made visible by placing a salient object on the top of it, allowing the mice to locate it using visual cues. To avoid spatial bias, the platform was positioned in a different quadrant from those used during the acquisition and retention phases. The time taken to reach the platform and the time spent in the different quadrants were recorded using a video camera (Sony CCD-IRIS, Tokyo, Japan) and computerized by video-tracking program (EthoVision XT 11.5, Noldus Information Technologies, Wageningen, The Netherlands).

### 2.7. Methylation Analysis

DNA extraction from hippocampal tissue was performed using the SPEDDTOOLS Tissue DNA Extraction Kit from Biotools (Madrid, Spain; Catalog 21.136-4195). The concentration and purity of the extracted DNA were measured after each extraction using the Nanodrop 2000 spectrophotometer (Thermo Fisher Scientific, Waltham, MA, USA). Global DNA methylation detection was then performed by the Methyl Flash^TM^ Global DNA Methylation (5-mC) ELISA Easy Kit (Colorimetric) from Epigentek (Farmingdale, NY, USA; Catalog P-1030-96). According to the instructions provided by the manufacturers, hippocampal samples were adjusted to 100 ng. Briefly, methylated DNA was detected using capture and detection antibodies against 5-methylcytosine (5-mC) and then quantified colorimetrically by reading the absorbance at 450 nm using the BioTek PowerWave XS2 microplate reader (BioTek Instruments, Winooski, VT, USA). The amount of methylated DNA is proportional to the measured optical density intensity. To calculate the percentage of methylated DNA a standard curve was used. Each sample was analyzed in duplicate.

### 2.8. Statistical Analysis

ApoE and C57BL/6J mice were analyzed separately according to sex and treatment. The genotype factor refers exclusively to comparison between *APOE* genotypes. Data were analyzed using the SPSS 26.0 program (IBPM Corp, Chicago, IL, USA). First, a repeated measures (RM) analysis of variance (ANOVA) was performed with sex, genotype, and treatment as the main factors to analyze significant differences in those variables that were evaluated over time. Similarly, the overall effects of sex, genotype, and treatment, as well as their interactions, were evaluated by a univariate two- or three-way ANOVA for variables analyzed only once. A one-sample *t*-test was also used to compare the results with a chance level of 15 s. Homogeneity of variance was assessed using Levene’s test to determine whether the data were parametric or nonparametric. To assess sex, genotype, and/or treatment differences, one-way ANOVA followed by Tukey’s *post hoc* test or two-sample *t*-test was used for parametric data, while the Kruskal–Wallis test or Mann–Whitney U-test were used for nonparametric data. A paired sample *t*-test was used to assess differences in the time spent in the TQ depending on the MWM phase. Statistical significance was set at *p* value < 0.05, and results are represented as mean value ± standard error of the mean (S.E.M).

## 3. Results

### 3.1. Body Weight Was Affected by Sex and Gestational Exposure to Low Doses of CPF

After weaning, the body weight of mice was monitored on PND 60 and during behavioral testing. For C57BL/6J mice, a two-way RM ANOVA (sex and treatment) indicated that animals increased body weight over time [F_3,36_ = 3.741, *p* = 0.019]. Furthermore, interactions between age and treatment [F_3,36_ = 3.669, *p* = 0.021] and between age and sex [F_3,36_ = 2.955, *p* = 0.045] were observed, indicating that CPF-treated animals showed a lower body weight at PND 60, while females consistently exhibited a lower weight across all evaluated ages (Supplementary Figure S1).

Similarly, apoE homozygous mice carrying the human *APOE* ε3 or ε4 alleles showed that body weight increased over time [F_3,80_ = 20.047, *p* < 0.001]. The RM ANOVA with sex, treatment, and genotype as the main factors indicated the following interactions: age × treatment [F_3,80_ = 2.827, *p* = 0.044], age × sex [F_3,80_ = 12.979, *p* < 0.001], and age × sex × genotype [F_3,80_ = 4.626, *p* = 0.005]. Thus, a lower body weight was observed at PND 60 following the exposure to prenatal CPF, and this persisted across all evaluated time points in females. Furthermore, males consistently exhibited higher body weight than females regardless of the *APOE* genotype, except for ε4 carriers at PND 60, where no significant differences were observed (Supplementary Figure S2).

### 3.2. Activity in the Light and Dark Test Was Mainly Influenced by Sex and Treatment

**Figure 2:**
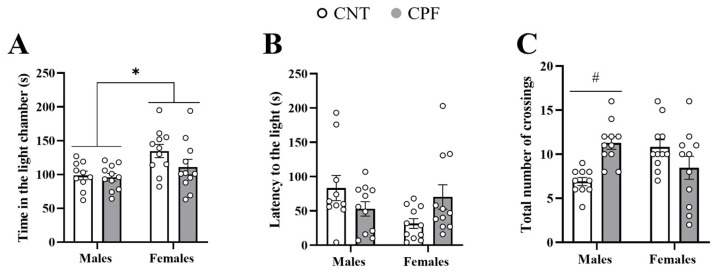
Light and dark test performed with C57BL/6J mice. Time spent in the light compartment (**A**), latency to first entry into the light compartment (**B**), and total number of crossings between the dark and light compartment (**C**) during the five minute test. Symbols indicate significant differences between sexes (*) and treatments (#) at *p* < 0.05.

The time spent in the light compartment, the first time that the animals cross from the dark to the light compartment (latency to the light), and the total number of crossings were evaluated to identify anxiety-like behaviors in both strains evaluated. A univariate two-way ANOVA with sex and treatment as main factors showed that C57BL/6J females spent more time in the light compartment than males [F_1,42_ = 8.597, *p* = 0.006] (Figure 2A). In addition, we found an interaction between sex and treatment in the analysis of the latency to enter to the light compartment [F_1,42_ = 5.999, *p* = 0.019] and the total number of crossings [F_1,42_ = 13.498, *p* = 0.001] (Figure 2B,C). Subsequent two-sample *t*-test analysis with the treatment as the independent variable showed a non-significant trend towards increased latency to enter the light compartment in C57BL/6J females treated with CPF [t_20_ = −2.023, *p* = 0.064], while C57BL/6J males treated with CPF increased the number of crossings [t_19_ = −4.471, *p* < 0.001] (Figure 2B,C), suggesting hyperactivity in males, especially in those treated with CPF.

**Figure 3:**
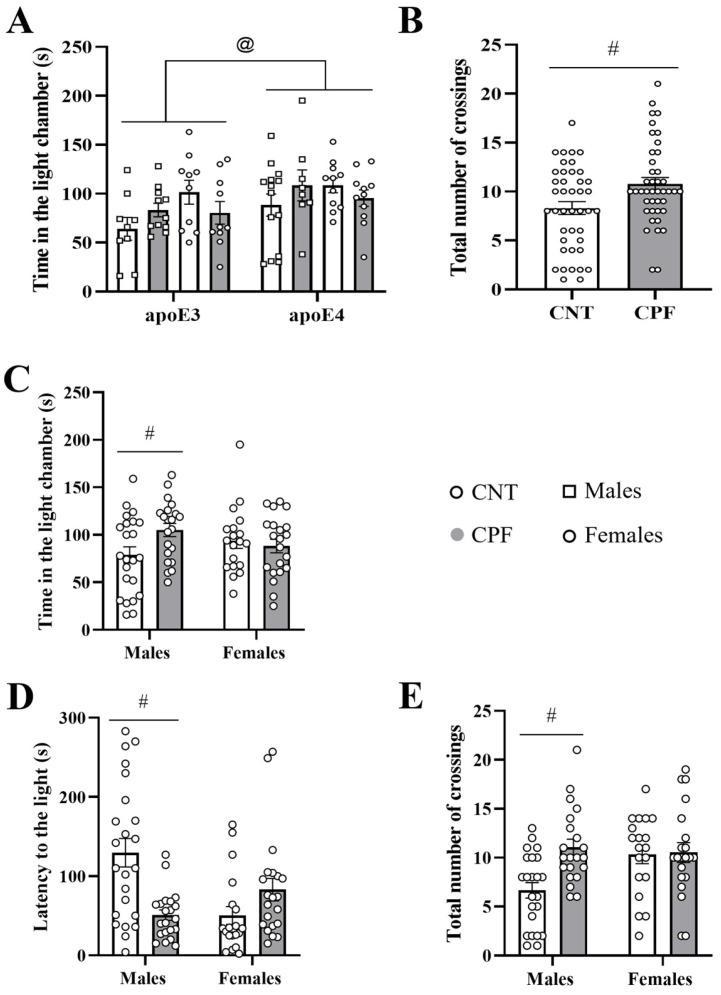
Light and dark test performed with apoE-TR mice. Time spent in the light compartment according to genotype (**A**) and total number of crossings according to treatment (**B**). Sex × treatment interaction in the time spent in the light compartment (**C**), latency to enter the light compartment (**D**) and total number of crossings between dark and light compartment (**E**) during the five minutes of the test. The symbol # and @ indicate significant differences between treatments and genotypes, respectively, at *p* < 0.05.

Regarding *APOE* genotype, the univariate three-way ANOVA analysis (sex, genotype, and treatment) showed an overall effect of genotype in the time that apoE mice spent in the light compartment [F_1,82_ = 5.223, *p* = 0.025] and a general effect of treatment in the total number of crossings [F_1,28_ = 6.932, *p* = 0.010] (Figure 3A,B). Thus, mice carrying the human *APOE* ε4 allele spent more time in the light compartment than apoE3 mice, while prenatal CPF exposure increased the number of crossings. Moreover, an interaction between sex and treatment was observed in the time spent in the light compartment [F_1,82_ = 5.468, *p* = 0.022], the total number of crossings [F_1,82_ = 4.307, *p* = 0.041], and the latency to enter the light compartment [F_1,82_ = 16.252, *p* < 0.001] (Figure 3C–E). Two-sample *t*-test analysis indicated that male mice treated with CPF took less time to switch compartments [t_41_ = 3.656, *p* = 0.001] and spent more time in the light compartment [t_41_ = −2.276, *p* = 0.028], crossing between the two compartments more times than the CNT group [t_41_ = −3.656, *p* = 0.001] (Figure 3C–E). These findings suggest that prenatal CPF exposure induced sex-specific alterations, particularly in males, which may be linked to increased hyperactivity and heightened exploratory activity.

### 3.3. Prenatal Exposure to CPF Did Not Affect Learning and Memory in C57BL/6J Mice

The time that the animals spent to find the platform was noted during the five days of the acquisition phase and analyzed using a RM ANOVA with the sex and treatment as the main variables. An overall effect of days was observed [F_4,35_ = 65.091, *p* < 0.001], indicating that all mice learned to reach the platform over the five days of training. In addition, a general interaction between sex and treatment was observed [F_1,38_ = 9.381, *p* = 0.004], indicating that male mice treated with CPF were the fastest to find the platform (Supplementary Figure S3A).

Short- and long-term memory were evaluated at 4 and 72 h, respectively, after the last trial of the acquisition phase. A univariate two-way ANOVA analysis (sex and treatment) did not show general effects of sex, treatment, or an interaction between them. However, all groups showed a good short- and long-term retention of the task, with all mice spending significantly more time in the TQ compared to the chance level of 15 s using a one-sample *t*-test (Supplementary Figure S3B).

In the third phase of the water maze test, we assessed the cognitive flexibility of the mice by relocating the escape platform to the opposite quadrant and analyzing their learning over two consecutive days. A two-way RM ANOVA (sex and treatment) indicated that animals learned the new location of the escape platform during the different trials [F_3,43_ = 20.443, *p* < 0.001]. Subsequently, the retention of the new platform location was performed 4 h after the last reversal trial. All groups learned the new location of the platform, with all mice spending more time in the new TQ. No effects of sex or treatment were observed (Supplementary Figure S3C).

Finally, the visual phase was performed to exclude impairments in vision, motor function, or escape motivation that could confound performance in any of the other MWM phases. The RM ANOVA (sex and treatment) did not show significant differences or interactions in the latency to reach the visible platform or in the distance swum along the four trials performed (Supplementary Figure S4). These results indicated that all animals were equally capable of locating a visible cue and exhibited comparable swimming abilities. Latency and total distance swam showed comparable patterns. For this reason, in Supplementary Figure S4, only latency is shown.

### 3.4. Long-Term Memory Was Impaired in apoE4 Mice Prenatally Exposed to CPF

**Figure 4:**
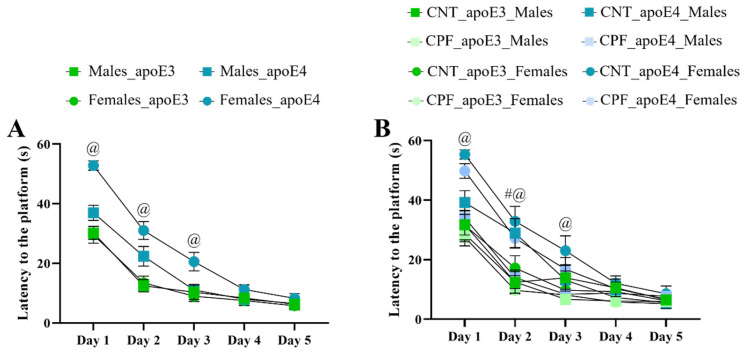
Acquisition in the MWM test. Latency to reach the escape platform over the five days of the training according to the interactions day × genotype × sex (**A**) and day × genotype × sex × treatment (**B**). Symbols # and @ indicate significant differences between treatment and genotype, respectively, at *p* < 0.05.

Learning performance during the acquisition phase of MWM was analyzed by a univariate three-way RM ANOVA (sex, treatment, and genotype). An overall improvement in the performance was observed throughout the acquisition sessions, as evidenced by a decrease in the time taken to find the escape platform [F_4,78_ = 176.608, *p* < 0.001]. The escape latency was also modified by the following interactions: day × genotype [F_4,78_ = 11.738, *p* < 0.001], day × sex [F_4,78_ = 3.012, *p* = 0.023] (Supplementary Figure S5), day × genotype × sex [F_4,78_ = 3.659, *p* = 0.009], and day × genotype × sex × treatment [F_4,78_ = 2.791, *p* = 0.032] (Figure 4). The interactions among day, genotype, and sex indicate that apoE4 females requiring more time to locate the hidden platform compared to apoE3 females during the first three days of training (day 1 and 2 (*p* < 0.001) and day 3 (*p* = 0.003)) consistently performed worse than apoE3 females during the first three days of the acquisition phase (Figure 4). Similarly, the interaction of day × genotype × sex × treatment (Figure 4) was analyzed by a three-way ANOVA with the Tukey *post-hoc* and indicated that non-treated apoE4-males showed poorer learning acquisition than CPF-treated male groups on day 2 (*p* = 0.018), while apoE4-females displayed the poorest performance up to day 3, regardless of treatment (day 1: CNT apoE4 vs. CNT apoE3 (*p* < 0.001) and CPF apoE3 (*p* < 0.001), CPF apoE4 vs. CNT apoE3 (*p* = 0.005) and CPF apoE3 (*p* = 0.001); day 2: CNT apoE4 vs. CNT apoE3 (*p* = 0.025) and CPF apoE3 (*p* < 0.001), CPF apoE4 vs. CNT apoE3 (*p* = 0.035) and CPF apoE3 (*p* < 0.001); day 3 CNT apoE4 vs. CNT apoE3 (*p* = 0.022) and CPF apoE3 (*p* = 0.031), CPF apoE4 vs. CNT apoE3 (*p* = 0.042)).

**Figure 5:**
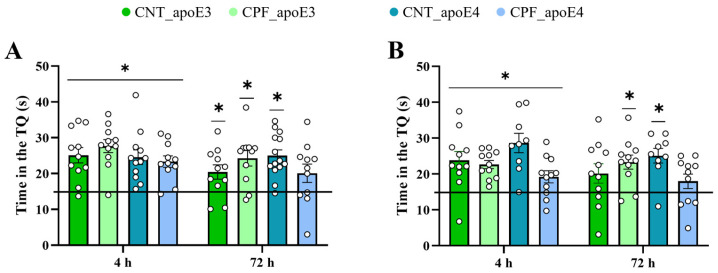
Retention in the MWM test. Time in the TQ in the two retention sessions (4 and 72 h) separately for males (**A**) and females (**B**). The symbol * indicates a performance significantly different from the chance level of 15 s at *p* < 0.05.

After acquisition, we evaluated the memory of the animals by removing the platform and analyzing the time that the mice spent in the TQ. These retention sessions were performed at 4 h (short-term) and 72 h (long-term) of the last acquisition trial. A one-sample *t*-test was used to analyze the time that the animal spends in the TQ in comparison with a chance level of 15 s (Figure 5A,B). Both apoE3 and apoE4 males and females showed a significant preference for the target quadrant 4 h after the last acquisition trial (apoE3 males: CNT [t_10_ = 4.718, *p* = 0.001], CPF [t_10_ = 6.977, *p* < 0.001]; apoE3 females: CNT [t_10_ = 3.618, *p* = 0.005], CPF [t_10_ = 6.648, *p* < 0.001] and apoE4 males: CNT [t_13_ = 5.639, *p* < 0.001], CPF [t_10_ = 5.162, *p* < 0.001]; apoE4 females: CNT [t_8_ = 5.043, *p =* 0.001], CPF [t_10_ = 2.480, *p* = 0.033]), while apoE4 males treated with CPF [t_10_ = 1.983, *p* = 0.075] and apoE3 CNT females [t_10_ = 1.894, *p* = 0.087] showed a non-significant trend for the TQ. apoE4 females prenatally treated with CPF showed no preference for the TQ [t_10_ = 1.464, *p* = 0.174], suggesting that treatment had a long-term detrimental effect in those mice carrying the human ε4 allele. All other groups showed a preference for the TQ (apoE3 males: CNT [t_10_ = 2.735, *p* = 0.021], CPF [t_10_ = 4.255, *p* = 0.002]; apoE3 female: CPF [t_10_ = 4.270, *p* = 0.002] and apoE4 males: CNT [t_13_ = 6.190, *p* < 0.001]; apoE4 females: CNT [t_8_ = 4.913, *p =* 0.001]) (Figure 5A,B).

**Figure 6:**
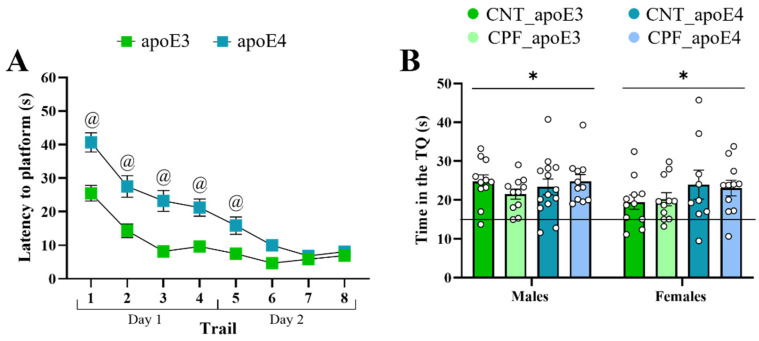
Reversal in the MWM test. Latency to reach the scape platform over the two days of the training (**A**). Time in the TQ 4 h after the last reversal trial (**B**). The symbol @ indicates significant differences between genotypes and an * indicates a performance significantly different from the chance level of 15 s at *p* < 0.05.

The reversal phase, in which the platform location is changed, is used to assess cognitive flexibility in mice. A univariate three-way RM ANOVA (sex, treatment and genotype) showed that animals learned the new location of the platform over the two-day sessions [F_7,75_ = 30.815, *p* < 0.001]. In addition, the following interaction was also observed: day × genotype [F_7,75_ = 4.022, *p* = 0.001] (Figure 6A) indicating a worse performance of the apoE4 mice during the first acquisition day. To further analyze these differences, we performed a two-sample *t*-test or Mann–Whitney U test, depending on the homogeneity of the samples. Significant differences were observed in all trials performed on day 1 (trial 1 (*p* < 0.001), trial 2 (*p* = 0.002), trial 3 (*p* < 0.001), and trial 4 (*p* < 0.001)) and the first two trials of day 2 (trial 1 (*p* = 0.027) and trial 2 [t_87_ = −2.785, *p* = 0.007]), indicating that apoE4 mice had more difficulty to reach the platform. The time that the mice spent in the TQ was then compared with a chance level of 15 s (Figure 6B). One-sample *t*-test showed a general preference for this quadrant by all the groups (apoE3 males: CNT [t_10_ = 5.575, *p* < 0.001], CPF [t_10_ = 4.988, *p* = 0.001]; apoE3 females: CNT [t_10_ = 2.391, *p =* 0.038], CPF [t_10_ = 3.103, *p* = 0.011]; apoE4 males: CNT [t_13_ = 4.148, *p* = 0.001], CPF [t_10_ = 5.495, *p* < 0.001]; apoE4 females: CNT [t_8_ = 2.399, *p* = 0.043], CPF [t_10_ = 4.001, *p* = 0.003]).

At the end of the MWM, the visual phase was performed. A RM ANOVA (sex, treatment and genotype) revealed a significant interaction of trial × sex × treatment for both latencies to reach the visible platform [F_3,79_ = 4.423, *p* = 0.006] and total distance swum [F_3,79_ = 3.222, *p* = 0.027]. Subsequently, the Mann–Whitney test indicated that CNT males exhibited a progressive increase in both latency and distance over the course of the trials (latency: trial 1 (*p* = 0.806), trial 2 (*p* = 0.051), trial 3 (*p* = 0.019), and trial 4 (*p* = 0.005); distance: trial 1 (*p* = 0.915), trial 2 (*p* = 0.043), trial 3 (*p* = 0.019), and trial 4 (*p* = 0.021)), suggesting reduced efficiency in locating the visible platform (Supplementary Figure S6A,B). As both latency and distance showed the same pattern of results, only latency is presented in the Supplementary Materials as a representative metric. To better analyze these effects, we performed a paired sample *t*-test with the time in the TQ during the visual vs. the time in the TQ during reversal and acquisition. Supplementary Figure S6C showed that CNT male mice spent significantly more time in the TQ where the platform was located during the reversal, rather than in the TQ where the platform was located during the visual phase (TQ visual vs. TQ reversal: trial 1 [t_24_ = −0.692, *p* = 0.495], trial 2 [t_24_ = −2.331, *p* = 0.028], trial 3 [t_24_ = −2.137, *p* = 0.043], and trial 4 [t_24_ = −2.773, *p* = 0.011]). This indicates a perseverative behavior and reliance on previously learned spatial strategies, even when visual cues were available. In contrast, all other significant differences observed were due to increased time spent in the TQ of the visual phase, suggesting appropriate task adaptation. Notably, this perseverative pattern was not observed during other MWM phases, highlighting that the effect is specific to the visual phase where spatial memory should be overridden by visual cues.

### 3.5. Prenatal Exposure to CPF Affects Total DNA Methylation in a Sex-Dependent Manner

Based on the results obtained in behavioral tests, we performed the analysis of global DNA methylation only on the hippocampus of apoE mice. Hippocampal samples were collected 24 h after the MWM test, considering the central role of this brain area in both anxiety-like behaviors and learning and memory. Given the exploratory nature of this analysis, we aimed to assess the potential of prenatal CPF exposure as an epigenetically active compound that may influence DNA methylation patterns in adulthood.

**Figure 7:**
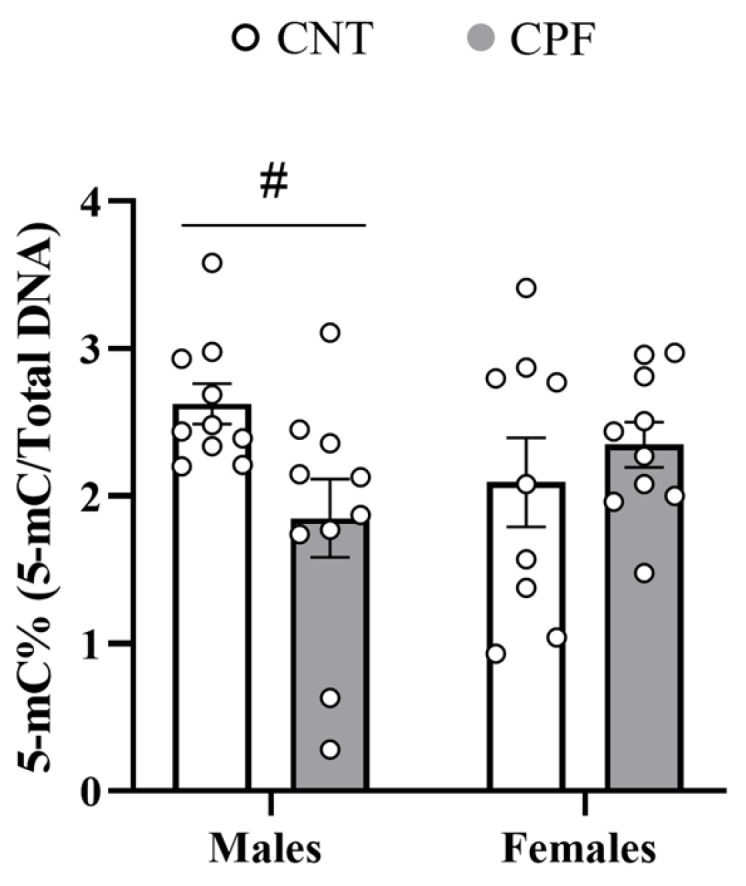
Global DNA methylation in apoE mice. The symbol # indicates significant differences between treatments at *p* < 0.05.

Statistical analysis using a univariate three-way ANOVA with sex, treatment, and genotype as the main variables indicated that prenatal exposure to CPF affects global DNA methylation depending on sex [F_1,38_ = 4.991, *p* = 0.033]. To further analyze this interaction, we performed a two-sample *t*-test with treatment as the independent variable, and significant hypomethylation in male mice prenatally treated with CPF [t_18_ = 2.594, *p* = 0.018] (Figure 7) emerged. In contrast, no significant differences were found between CNT and treated females [t_17_ = −0.750, *p* = 0.468], supporting a sex-specific effect of CPF exposure (Figure 7).

## 4. Discussion

An increasing body of evidence indicates that environmental exposures from the prenatal stage onward constitute modifiable risk factors for a wide range of neurodevelopmental and neurodegenerative disorders. In this study, we investigated the long-term detrimental effects of prenatal exposure to CPF, as well as the influence of the *APOE* genotype on anxiety and learning and memory using a transgenic mouse model carrying the human *APOE* ε3 and ε4 alleles. Moreover, we evaluated the potential effects of CPF as a methylating agent on global DNA methylation in a transgenic mouse model carrying most common *APOE* genotype.

Specifically, male and female C57BL/6J, apoE3 and apoE4 mice that were indirectly treated with low doses of CPF during gestation were evaluated at the age of three months. Light and dark and MWM tests were performed to investigate anxiety-like behavior and early learning and memory impairments. Briefly, body weight was lower in female mice and in those treated with CPF, especially on PND 60. In terms of anxiety-like behavior, although C57BL/6J males spend less time in the light compartment, prenatal CPF exposure increased their activity, as evidenced by an increased number of crossings in that sex. In the same line, only transgenic male mice carrying the human *APOE* ε4 or ε3 allele were affected in. Indeed, mice that were prenatally dosed with CPF spent more time in the light compartment, showed shorter latencies to enter it, and carried out more crossings, indicating increased activity, while females were unaffected. Moreover, a general effect of genotype was observed, with *APOE* ε4 spending more time in the light compartment. Regarding the MWM test, all mice successfully acquired the task by day 5, but females carrying the *APOE* ε4 allele performed worse during the first three days. Moreover, both short- and long-term memory were impaired in apoE4 females that were prenatally treated with CPF. Together with these behavioral results, we found changes in the methylation pattern in males dosed with CPF.

Exposure to CPF has been related to an increased incidence of obesity in recent decades, due to its obesogenic effects [59]. By contrast, our results showed that prenatal CPF exposure during the late gestational period reduced body weight on PND 60. However, this difference was no longer evident at later ages, as body weight normalized over time across groups. A previous study conducted in our laboratory found that exposure to CPF from GD 12 to 18 at a dose below the toxicity threshold (1 mg/kg) produced a decrease in body weight that persisted until weaning [58]. Another study involving a longer exposure period (from GD 7 to the end of lactation (PND 21)) and a higher dose of CPF (2.5 mg/kg/day) in rats, indicated that male offspring had a higher body weight than the CNT group after weaning, while the weight of females remained unchanged [60]. Along these lines, postnatal exposure to CPF has been strongly associated with increased body weight in both male and female rodents [53,61,62]. This increase was most evident in 5-month-old apoE3 and apoE4 animals treated during the pre-weaning period and then given a further dose of 2 mg/kg/day of CPF for 15 days [53]. Thus, the data suggest that the impact of CPF on body weight depends critically on the timing and duration of exposure, with more pronounced obesogenic effects observed when the exposure occurs during postnatal development. However, further research is needed to investigate the consequences of repeated CPF exposure across the lifespan and to determine what underlying mechanisms exist at each stage of life that may lead to weight gain in certain times and weight loss at others, or that may modify the long-term metabolic effects associated with early life exposure.

Notable differences were observed in anxiety-related and cognitive behavior. Anxiety disorders are becoming increasingly prevalent in the population due to the current lifestyle. Javaid et al. [63] revealed a global prevalence of anxiety disorders around 4.05%, with a peak at 35–39 years of age, being aggravated by exposure to environmental toxicants or by genetic polymorphisms. The light and dark test mimics natural rodent behavior, and rodents tend to avoid brightly lit spaces and prefer the safety of dark environments. However, rodents are curious and may explore the lighted area [64]. Regarding C57BL/6J mice, CPF exposure has a sex-dependent effect, increasing the number of inter-compartment crossings only in males, suggesting enhanced activity. Similar effects have been observed in apoE mice, where prenatal pesticide exposure led to an increased number of crossings and the time spent in the light compartment in apoE males. Simultaneously, these animals also showed reduced latency in switching from the dark to the light compartment. This suggests that apoE males exhibit a hyperactive behavioral profile following prenatal pesticide exposure characterized by increased exploratory behavior and reduced hesitation. Although some studies have investigated the interaction between CPF and *APOE* genotype in anxiety-related behaviors, sex differences in this context remains unexplored [65]. However, earlier studies by Ricceri et al. [23,66] have reported increased activity following CPF exposure. Specifically, CD-1 male mice exposed to 3 mg/kg/day of CPF between PND 11 and 14 showed hyperactivity during the weaning period (PND 25) [66]. Subsequent studies confirmed that gestational exposure to CPF at doses of 3 or 6 mg/kg/day produced a pronounced hyperactivating effect on adult male CD-1 mice. This heightened activity was maintained in those animals that had received the higher prenatal dose and were later re-dosed with CPF (1 mg/kg/day) during the postnatal period (from PND 11 to 14) [23]. Thus, gestational exposure to CPF leads to long-lasting and sex-dependent alterations resulting in exaggerated activity levels, with male mice exhibiting a greater vulnerability.

Regarding *APOE* polymorphism, animals carrying the *APOE* ε4 allele spent more time in the light compartment compared to *APOE* ε3 carriers, a pattern also observed in C57BL/6J females. Although this behavior might initially suggest reduced anxiety-like responses, the interpretation becomes more complex when considering that increased time in the light compartment could also reflect a lack of movement or a freezing response rather than active exploration. Similarly, the time spent in the light compartment could reflect altered activity levels, highlighting the importance of considering locomotor and exploratory behaviors when interpreting outcomes in this test. In supporting this, a previous study found that animals carrying the human *APOE* ε4 allele showed a higher number of freezing episodes in the open area of the elevated plus maze test compared to *APOE* ε2 and ε3 carriers, as well as lower activity and number of crossings or head-dips [67]. In addition, lower distance and velocity in the central area of an open field test were observed in apoE4 mice [58]. Sex differences in anxiety-like behaviors have also been reported by Börchers et al. [68], who found that 23-week-old female rats spent significantly more time in open or anxiogenic areas of both the elevated plus maze and the open field tests. These findings reinforce the idea that both sex and genotype significantly influence the interpretation of anxiety-related behaviors and that multiple variables must be considered for a rigorous and accurate analysis.

Given that the *APOE* genotype has a strong association with AD, particularly the ε4 allele. This study assessed the effects of *APOE* polymorphism and CPF pesticide exposure on spatial learning and memory using the MWM test, which relies on rodents’ natural aversion to water and their preference to escape [69,70]. C57BL/6J mice showed a good acquisition of the task over the five days, with males treated prenatally with CPF finding the platform more quickly. Furthermore, all animals remembered the location of the platform in all cases. Similarly, humanized apoE mice learned the task over the five days; however, apoE4 females exhibited slower performance, especially during the first three days. Homozygous apoE4 mice also showed both short- and long-term memory impairments, which were further aggravated by prenatal CPF exposure. Moreover, mice with the *APOE4* genotype performed the worst in remembering the platform’s new location. Previous studies using an object recognition test, indicated that 2-month-old *APOE* ε4 carriers treated with CPF (1 mg/kg/day) during a defined period of postnatal phase (from PND 10 to 15) showed impairments in recognition memory, evidenced by reduced discrimination between familiar and novel object [71]. Similarly, exposure to the same dose and during the same period in 9-month-old apoE4 mice revealed that CPF-treated males showed irregular performance, while apoE4 females had impaired recall. This impairment was further worsened by postnatal CPF exposure, particularly at the end of the MWM task [51]. These findings support the synergistic effect of the *APOE4* genotype and CPF exposure during sensitive windows of brain development on later cognitive decline, with more severe consequence in females. This behavioral profile may be linked to developmental AChE inhibition by CPF, which dysregulates cholinergic signaling. In apoE4 mice, this disruption is exacerbated by a pre-existing lower cholinergic density and reduced AChE levels, leading to the observed cognitive deficits [72]. Therefore, when assessing the neurobehavioral effects of toxic agents in both preclinical models and epidemiological studies, the time of toxic exposure, genetic profile and sex are key factors to consider. In conclusion, these results provide evidence supporting that the *APOE* ε4 allele not only predispose or represents a distinct genetic form to the development of neurodegenerative pathologies such as AD [73,74] but also increases sensitivity to environmental factors such as pesticides.

Sex-dependent differences observed in learning and memory were also observed in global DNA methylation, but in this case, CPF-treated males showed lower levels. These alterations in the pattern of DNA methylation resulted in the dysregulation of gene networks associated with neurodevelopmental and neurodegenerative disorders, which can lead to long-term cognitive and behavioral alterations [75,76]. Notably, in our case, males prenatally exposed to CPF also exhibited more pronounced behavioral effects related to hyperactivity. The hippocampus plays a central role in regulating emotions and stress responses, especially in males [77]. Thus, reduced global methylation could increase transcription of activity-dependent and synaptic genes—such as neurotrophic factors, glutamatergic receptor subunits, dopaminergic signaling genes, or stress regulators—thereby providing mechanistic explanation for the hyperactivity observed in CPF-exposed males, a finding that aligns with human studies linking exposure to OP pesticides with genome-wide methylation modifications in several genes [78,79]. To date, only one study has directly examined the effect of CPF exposure in relation to the *APOE* genotype on DNA methylation levels, highlighting that this is an unexplored area of research that is attracting increasing interest [55]. Unlike our study, Guardia-Escote et al. [55] investigated specific methylation patterns of genes involved in feeding control in the hypothalamus. Male apoE mice treated with a low dose of CPF from PND 10 to 15 showed higher methylation levels in genes such as *Pomc* or *Lepr*, while the mean methylation of *Npy* was decreased. These findings are consistent with previous studies that have suggested males may be more vulnerable to epigenetic reprogramming during critical periods of development [80]. As highlighted by Kundakovic and Tickerhoof [81], male and female brains exhibit different epigenetic patterns shaped by genetic and hormonal factors that modulate the effect of environmental toxin exposure on neurodevelopment and behavior.

In summary, this study highlights the significant impact of the interaction between genetic factors such as the *APOE* polymorphism and prenatal environmental exposure to CPF on neurobehavioral development, with clear sex-dependent effects. The presence of two *APOE* ε4 copies was associated with spatial learning and memory impairments, which were further exacerbated by CPF exposure. Prenatal CPF exposure also led to a reduction in body weight, increased the activity in the light and dark test, and induced hypomethylation in males. These epigenetic changes suggest that CPF may act as a modulatory factor of the epigenome. Together, these findings reveal that early prenatal exposure, limited to embryonic development, is sufficient to trigger long-lasting neurobehavioral alterations. Overall, this research emphasizes the importance of considering genetic background, timing of exposure, and sex as key variables when assessing the neurobehavioral effects of toxic agents. Furthermore, it opens promising avenues for investigating epigenetic mechanisms as mediators of gene–environment interactions, which impact cognitive and emotional outcomes.

## Figures and Tables

**Table 1:** Total number of animals per group in the behavioral test and biochemical analysis.

Strain	Treatment	Behavioral Test		Methylation Analysis	
		Males	Females	Males	Females
C57BL/6J	CNT	11	11	-	-
	CPF	11	11	-	-
apoE3	CNT	11	11	5	5
	CPF	11	11	5	5
apoE4	CNT	14	9	5	4
	CPF	11	11	5	5

CNT: control; CPF: chlorpyrifos.

## Data Availability

The raw data supporting the conclusions of this article will be made available by the authors on request.

## Supplementary Materials

The following supporting information can be downloaded at: https://www.mdpi.com/article/10.3390/toxics14030212/s1. Figure S1. Body weight in C57BL/6J mice. CPF-treated mice showed overall reduced body weight compared to CNTs (age × treatment [F_3,36_ = 3.669, *p* = 0.021]), with a significant difference observed at PND 60 [t_40_ = 3.061, *p* = 0.004]; Figure S2. Body weight in apoE-TR mice. CPF-treated mice showed overall reduced body weight compared to CNTs (age × treatment [F_3,80_ = 2.827, *p* = 0.044]), with a significant difference observed at PND 60 [t_89_ = 2.613, *p* = 0.011]; Figure S3. Retention phase of the MWM test in C57BL/6J mice. A general sex × treatment interaction was found [F_1,38_ = 9.381, *p* = 0.004], with male mice exposed to CPF being faster to reach the platform than CNT group [t_20_ = 4,726, *p* < 0.001]; Figure S4. Visual phase of the MWM test in C57BL/6J mice. The latency to reach the visible platform did not differ significantly across trials [F_3,38_ = 0.652, *p* = 0.586], nor were there significant interactions with sex, [F_3,38_ = 1.241, *p* = 0.308], treatment [F_3,38_ = 1.040, *p* = 0.386] or their combination (trial × sex × treatment) [F_3,38_ = 1.363, *p* = 0.269], indicating intact visual and motor capabilities between groups; Figure S5. Acquisition in the MWM test. A day × genotype interaction was found [F_4,78_ = 11.738, *p* < 0.001], with homozygous mice for the human APOE ε4 allele showed slower learning than APOE ε3 carriers. Mann-Whitney U test revealed that this difference was significant during the first three days of the test (day 1 and 2 (*p* < 0.001) and day 3 (*p* = 0.008)); Figure S6. Visual phase of the MWM test in apoE-TR mice. A sex × treatment interaction was observed [F_3,79_ = 4.423, *p* = 0.006]. The U de Mann-Whitney test indicated that CNT males showed a progressive increase through the performance of trials (trial 1 (*p* = 0.806), trial 2 (*p* = 0.051), trial 3 (*p* = 0.019) and trial 4 (*p* = 0.005)).

## Abbreviations

The following abbreviations are used in this manuscript:

ACh	Acetylcholine
AChE	Acetylcholinesterase
AD	Alzheimer disease
ANOVA	Analysis of variance
APOE	Apolipoprotein E
CNS	Centra nervous system
CNT	Control
CPF	Chlorpyrifos
DDT	Dichlorodiphenyltrichloroethane
GD	Gestational day
MWM	Morris water maze
OP	Organophosphate
PND	Postnatal day
RM	Repeated measures
TQ	Target quadrant
TR	Target replacement
