# Supplementary material for: Sex and APOE ε4 Allele Shape Behavioral and Epigenetic Susceptibility to Prenatal Chlorpyrifos Exposure in Mice
Source: Toxics. 2026 Feb 28;14(3):212. doi: 10.3390/toxics14030212 (PMC13029873; doi:10.3390/toxics14030212)
Supplement: Supplementary file 1 [file toxics-14-00212-s001.zip › toxics-4131985-supplementary.pdf]

Supplementary Material

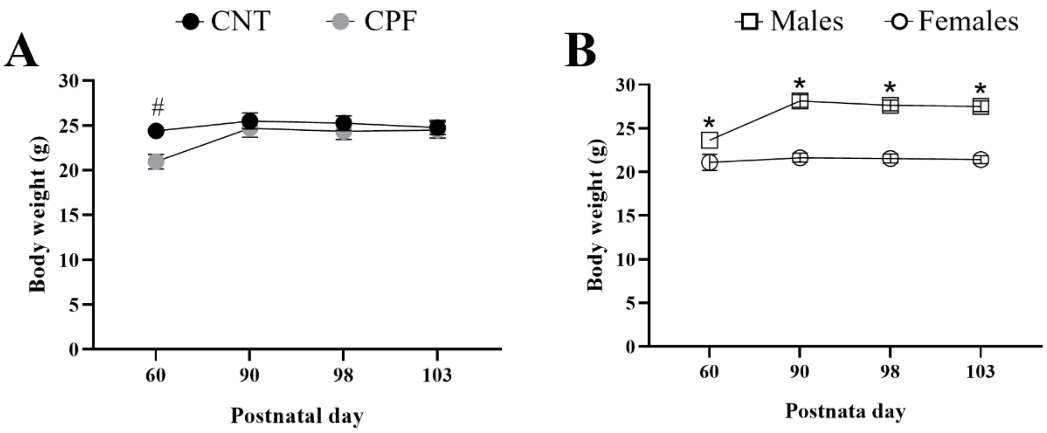

Figure S1. Body weight in C57BL/6J mice. CPF-treated mice showed overall reduced body weight compared to CNTs (age x treatment [$F_{3,36}$=3.669, p=0.021]), with a significant difference observed at PND 60 [t_{40}=3.061, p=0.004] (**A**). A general effect of sex (age x sex [$F_{3,36}$=2.955, p=0.045]) was observed, with females displaying lower body weight during all the evaluation timepoints (PND 60 [t_{40}=2.189, p=0.034], PND 90 (p<0.001), PND 98 [t_{40}=7.542, p<0.001] and PND 103 [t_{40}=7.893, p<0.001]) (**B**). The symbol # indicates significant differences between treatments, while an * indicates significant differences between sexes at p<0.05.

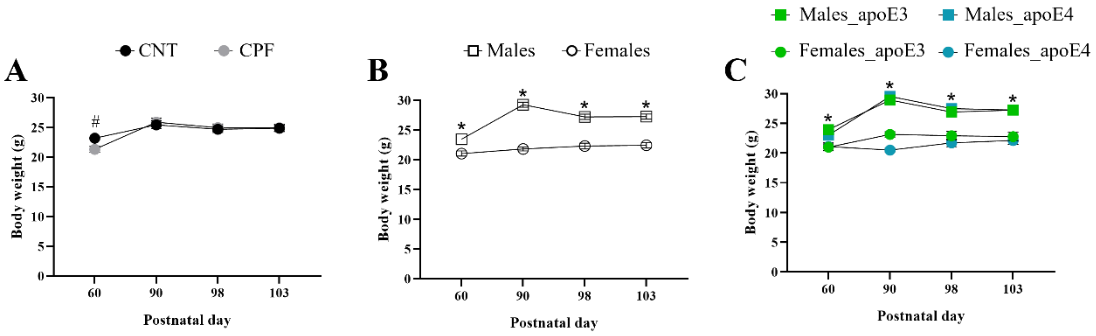

Figure S2. Body weight in apoE-TR mice. CPF-treated mice showed overall reduced body weight compared to CNTs (age x treatment [$F_{3,80}$=2.827, p=0.044]), with a significant difference observed at PND 60 [t_{89}=2.613, p=0.011] (**A**). A general effect of sex (age x sex [$F_{3,80}$=12.979, p<0.001]) was observed, with females displaying lower body weight across all evaluated timepoints (PND 60 [t_{89}=3.445, p=0.001], PND 90 (p<0.001), PND 98 [t_{88}=7.499, p<0.001] and PND 103 [t_{88}=7.538, p<0.001]) (**B**). A significant age x sex x genotype interaction [$F_{3,80}$=4.626, p=0.005] was found, indicating that sex differences varied by genotypes. While apoE3 male mice showed higher body weight than females at PND60 [t_{42}=3.266, p=0.002], PND 90 (p<0.001), PND 98 [t_{41}=4.029, p<0.001] and PND 103 [t_{41}=4.560, p<0.001], apoE4 mice similar sex differences were observed at PND 90 (p<0.001), PND 98 [t_{45}=6.672, p<0.001] and PND 103 [t_{45}=6.081, p<0.001], but not at PND 60 [t_{45}=1.801, p=0.079] (**C**). The symbol # indicates significant differences between treatments, while an * indicates significant differences between sexes at p<0.05.

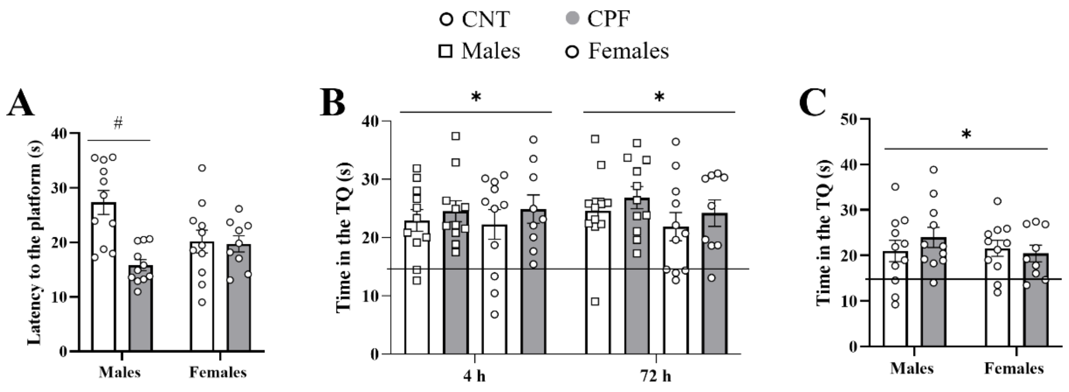

Figure S3. Retention phase of the MWM test in C57BL/6J mice. A general sex x treatment interaction was found [$F_{1,38}$=9.381, *p*=0.004], with male mice exposed to CPF being faster to reach the platform than CNT group [t20=4,726, p<0.001] (**A**). A one-way t-test analysis showed a general preference for the TQ at 4 and 72 h after the last acquisition session (4 h males: CNT [t_{10}=4.250, *p*=0.002], CPF [t_{10}=5.281, *p*<0.001]; 4 h females: CNT [t_{10}=2.820, *p*=0.018], CPF [t_{10}=4.077, *p*=0.004]; 72 h males: CNT [t_{10}=4.597, *p*=0.001], CPF [t_{10}=6.228, *p*<0.001]; 72 h females: CNT [t_{10}=2.849, *p*=0.017]; CPF [t_{10}=4.055, *p*=0.004]) (**B**). Similar preference for the TQ was observed after the last reversal trial (males: CNT [t_{10}=2.554, *p*=0.029], CPF [t_{10}=4.018, *p*=0.002]; females: CNT [t_{10}=3.760, *p*=0.004], CPF [t_{10}=2.969, *p*=0.018]) (**C**). The symbol # indicates significant differences between treatments, while an * indicates a significantly different performance from the chance level of 15 s at *p*<0.05.

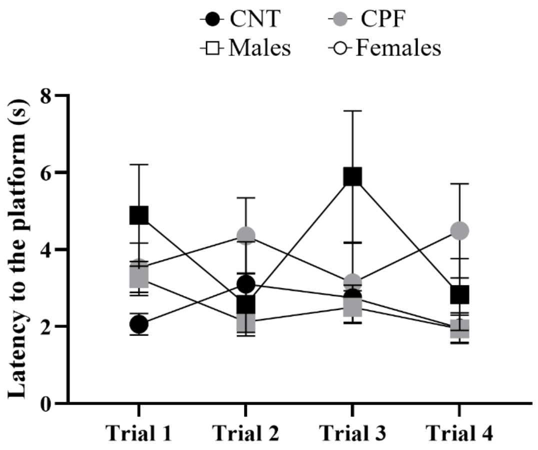

Figure S4. Visual phase of the MWM test in C57BL/6J mice. The latency to reach the visible platform did not differ significantly across trials [$F_{3,38}$=0.652, p=0.586], nor were there significant interactions with sex, [$F_{3,38}$=1.241, p=0.308], treatment [$F_{3,38}$=1.040, p=0.386] or their combination (trial x sex x treatment) [$F_{3,38}$=1.363, p=0.269], indicating intact visual and motor capabilities between groups.

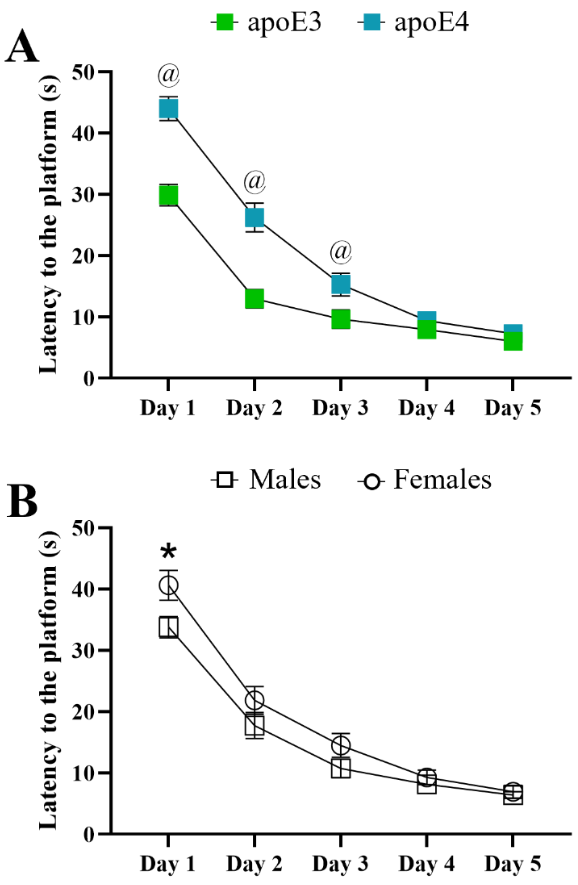

Figure S5. Acquisition in the MWM test. A day x genotype interaction was found [$F_{4,78}$=11.738, p<0.001], with homozygous mice for the human *APOE* ε4 allele showed slower learning than *APOE* ε3 carriers. Mann-Whitney U test revealed that this difference was significant during the firsts three days of the test (day 1 and 2 (p<0.001) and day 3 (p=0.008)) (**A**). A day x sex interaction was observed [$F_{4,78}$=3.012, p=0.023], with female mice showed more difficulty than males in performing the task, especially on the first day of the test (p=0.016) (B). The symbols @ and * indicate significant differences between genotypes and sex, respectively, at p<0.05.

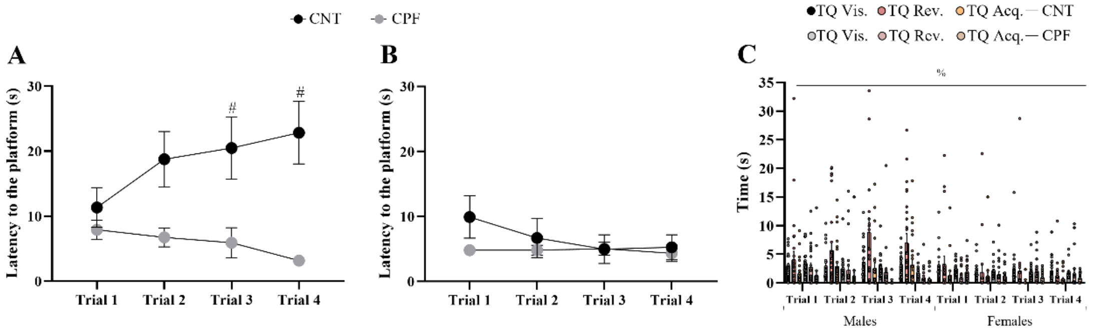

Figure S6. Visual phase of the MWM test in apoE-TR mice. A sex x treatment interaction was observed [$F_{3,79}$=4.423, p=0.006]. The U de Mann-Whitney test indicated that CNT males showed a progressive increase through the performance of trials (trial 1 (p=0.806), trial 2 (p=0.051), trial 3 (p=0.019) and trial 4 (p=0.005)) (**A**), while no significant differences were observed in females (trial 1 (p=0.420), trial 2 (p=0.147), trial 3 (p=0.089) and trial 4 (p=0.473)) (**B**). A paired sample t-test comparing TQ of the visual phase with TQ of the reversal and acquisition found

that CNT males spent more time in the reversal TQ than the visual TQ (TQ visual *vs* TQ reversal (CNT): trial 1 [t_{24}=-0.692, p=0.495], trial 2 [t_{24}=-2.331, p=0.028], trial 3 [t_{24}=-2.137, p=0.043] and trial 4 [t_{24}=-2.773, p=0.011]), while the other differences observed were due to increased time in the visual TQ (males TQ visual *vs* TQ acquisition (CNT): trial 1 [t_{24}=2.647, p=0.014], trial 2 [t_{24}=-0.045, p=0.965], trial 3 [t_{24}=1.423, p=0.168] and trial 4 [t_{24}=-0.254, p=0.802]; males TQ visual *vs* TQ reversal (CPF): trial 1 [t_{21}=0.908, p=0.374], trial 2 [t_{21}=0.194, p=0.848], trial 3 [t_{21}=1.455, p=0.160] and trial 4 [t_{21}=2.116, p=0.046]; males TQ visual *vs* TQ acquisition (CPF): trial 1 [t_{21}=4.443, p<0.001], trial 2 [t_{21}=3.192, p=0.004], trial 3 [t_{21}=6.176, p<0.001] and trial 4 [t_{21}=5.790, p<0.001]; females TQ visual *vs* TQ reversal (CNT): trial 1 [t_{19}=-0.087, p=0.931], trial 2 [t_{19}=0.007, p=0.995], trial 3 [t_{19}=0.628, p=0.537] and trial 4 [t_{19}=2.907, p=0.009]; females TQ visual *vs* TQ acquisition (CNT): trial 1 [t_{19}=2.319, p=0.032], trial 2 [t_{19}=1.422, p=0.171], trial 3 [t_{19}=3.231, p=0.004] and trial 4 [t_{19}=3.630, p=0.002]; females TQ visual *vs* TQ reversal (CPF): trial 1 [t_{21}=4.982, p<0.001], trial 2 [t_{21}=1.050, p=0.306], trial 3 [t_{21}=-0.142, p=0.889] and trial 4 [t_{21}=0.300, p=0.767]; females TQ visual *vs* TQ acquisition (CPF): trial 1 [t_{21}=2.618, p=0.016], trial 2 [t_{21}=2.686, p=0.014], trial 3 [t_{21}=3.835, p=0.001] and trial 4 [t_{21}=3.589, p=0.002];)) (**C**). Symbols # and % indicate significant differences between treatments and TQ, respectively, at p<0.05.
